# High serum IL-17A is associated with bone destruction in newly diagnosed multiple myeloma patients

**DOI:** 10.3389/fonc.2022.936670

**Published:** 2022-08-31

**Authors:** Mengmeng Dong, Jinna Zhang, Qingxiao Chen, Donghua He, Haimeng Yan, Gaofeng Zheng, Xiaoyan Han, Jingsong He, Zhen Cai

**Affiliations:** ^1^ Bone Marrow Transplantation Center, The First Affiliated Hospital, School of Medicine, Zhejiang University, Hangzhou, China; ^2^ Institute of Hematology, Zhejiang University, Hangzhou, China

**Keywords:** IL-17A, multiple myeloma, bone destruction, best overall therapeutic effect, pfs, os, light chain type, IgA

## Abstract

**Background:**

Multiple myeloma (MM) is a malignant proliferative disease of the blood system, characterized by the abnormal growth of clonal plasma cells in the bone marrow. The bone marrow microenvironment (BMM) is highly critical in the pathological process of MM. Many studies have shown that serum interleukin-17A (IL-17A) plays a key role in various infectious diseases, autoimmune diseases, and cancers. However, more clinical studies need to be performed to further prove the influence of serum IL-17A levels on multiple myeloma patients.

**Methods:**

Among a total of 357 participants in our institution’s MM cohort, 175 were eligible for the retrospective study. Multivariate regression models adjusted by potential confounding factors, the violin plots, the generalized additive model and smooth curve fittings, receiver operating characteristic (ROC) curve, and Kaplan–Meier (K-M) curve analysis were applied to the research.

**Results:**

A total of 175 patients with newly diagnosed MM were enrolled in this study. The multivariate linear regression analysis showed that serum IL-17A level in MM patients correlated with the degree of bone lesions and fracture incidence (fully adjusted model, p_bone lesion_ < 0.0001, p_fracture_ < 0.0001). The violin plot showed that MM patients with higher serum IL-17A levels had more severe bone lesions and higher fracture incidence than those with lower serum IL-17A levels. A total of 171 patients were included in the study of the relationship between serum IL-17A and best overall effect (BOE). We found that serum IL-17A levels were independently related to the best inductive therapeutic efficacy (fully adjusted model, p = 0.037), and the relationship was especially obvious in the light chain group (fully adjusted model, p = 0.009) and IgA group (fully adjusted model, p = 0.0456). It could be deduced from the smooth curve that the higher the serum IL-17A level, the worse the BOE (p = 0.0163). The ROC prediction curve suggested that serum IL-17A could predict the BOE to a certain extent (area under the curve (AUC) = 0.717, p = 0.0327). A total of 148 MM patients were observed in the longitudinal study of the relationship between serum IL-17A and progression-free survival/overall survival (PFS/OS). The K-M curve analysis indicated that serum IL-17A levels in MM patients were not significantly correlated with PFS and OS. However, in the light chain subgroup, MM patients with high serum IL-17A had worse PFS (p = 0.015) and OS (p = 0.0076) compared to those with low serum IL-17A. In the IgA type subgroup, the higher IL-17A level was related to worse OS (p = 0.0061).

**Conclusion:**

This retrospective study found that higher levels of serum IL-17A were independently correlated with higher severity of bone disease and fracture incidence in newly diagnosed MM patients. High serum IL-17A level was related to poor best overall efficacy in the light chain type. High serum IL-17A was also associated with poor PFS and OS in the light chain type and OS in the IgA type subgroup.

## Background

Multiple myeloma (MM) is a malignant proliferative disease of the hematological system characterized by the abnormal growth of clonal plasma cells in the bone marrow. These cells secrete monoclonal immunoglobulin or its fragments (M protein), resulting in damage to tissues and organs (1). The development of treatment options in the past 20 years has greatly improved the median survival of MM patients, but MM is still incurable (2). The bone marrow microenvironment (BMM) provides an immunosuppressive milieu for MM oncogenesis and tumor progression, which is critical in the pathological process of MM (3). As an important part of the BMM, various cytokines affect cell proliferation, drug resistance, and immune escape in MM (4).

The interleukin-17 (IL-17) family of cytokines includes IL-17A-IL-17F, which are mainly secreted by CD4+T helper 17 (Th17) cells, and they can also be derived from γδ T cells, CD8+αβ T cells, natural killer cells, and innate lymphoid cells (5). Recently, IL-17A has been the subject of the most extensive and in-depth research within the IL-17 family. IL-17A can resist extracellular bacterial and fungal infections (6, 7). At the same time, in many pathological processes, especially in the process of the chronic and continuous immune response, IL-17A can cause autoimmune diseases and damage tissue structures (8, 9). IL-17A is a key biomolecule in various infectious diseases, inflammatory disorders, autoimmune diseases, and cancers. Some studies suggest that IL-17A also promotes bone loss (10–13). In several cancers, including colon cancer (14), lung adenocarcinoma (15), liver cancer (16), skin malignant tumors (17), and pancreatic cancer (18), IL-17A also affects the therapeutic efficacy and tumor prognosis (19).

There have been basic studies on IL-17A promoting the occurrence of MM bone disease, promoting the proliferation of MM cells, increasing the viability of MM cells, and inhibiting immune function, although the mechanism of further action is still unclear (20–22). Noonan et al. found that the Th17 cytokine profile is related to bone disease, especially IL-17 presence in the bone marrow, and its level has a good correlation with the severity of bone destruction (20). It was also reported that in MM, IL-17A is related to angiogenesis (23). In terms of clinical research, some articles have suggested that MM patients have higher levels of IL-17 than normal controls and higher levels of IL-17A in advanced stages (19), and the increased ratio of IL-17:IL-27 in the bone marrow had worse progression-free survival (PFS) period (24). Genetic polymorphisms of IL-17A may affect disease severity, bone lesions, and extramedullary conditions in MM (25). Th17 cells are associated with skeletal diseases, and the anti-IL-17A monoclonal antibody (AIN457) can inhibit the clonal formation of MM cells (26). The above articles all observed small clinical samples (four patients to dozens of patients), their conclusions about the relationship between TH17/IL-17 and MM prognosis are not uniform, and there was no article that studied bone disease, treatment efficacy, and PFS/overall survival (OS) in the same cohort. A clearer relationship between IL-17A levels and bone disease, treatment efficacy, and PFS/OS needs to be explored/proven by more samples and more comprehensive clinical studies.

Therefore, we conducted this retrospective study to determine the relationship between serum ILα17A levels and bone lesions. In addition, we studied the role of serum IL-17A levels on the therapeutic effect and PFS/OS.

## Methods

In this single-center retrospective study, we collected the clinical data of newly diagnosed MM patients of the First Affiliated Hospital of Zhejiang University, School of Medicine, from May 2013 to May 2018. The diagnosis of MM was based on International Myeloma Working Group (IMWG) standards. The exclusion criteria were as follows: 1) lack of pre-chemotherapy clinical data and calcium or albumin values; 2) history of severe infection, kidney disease, liver disease, or autoimmune disease; 3) history of other solid tumors; and 4) lost to follow-up in the longitudinal study, resulting in the lack of PFS and OS data.

Our study was approved by the Ethics Committee of the First Affiliated Hospital of Zhejiang University (reference number: IT20210609A).

### Data collection

We electronically retrieved the baseline demographic and clinical data from the medical records of the general hospital registry and conducted a retrospective review. For MM patients admitted to the hospital multiple times, only the first set of observation data was used as baseline data.

The following indicators were evaluated: 1) demographic characteristics, including age, gender, hypertension, and diabetes); 2) laboratory data at initial diagnosis, including hemoglobin, serum creatinine, albumin, globulin, immunoglobulin, serum/urine light chain protein, IL-2, IL-4, IL-6, IL-10, TNF-α, IFN-γ, IL-17A, bone lesion, and fracture; 3) multiple myeloma treatment regimen; 4) best overall effect (BOE) within four cycles of induction therapy; 5) and PFS (referring to the time from the initial diagnosis to the first occurrence of disease progression or death from any cause) time within 30 months and OS referring to the time from the initial diagnosis to death from any cause) within 30 months.

### Investigation of study outcomes

First, we conducted a cross-sectional study to evaluate the relationship between serum IL-17A levels and bone destruction. The presence of bone destruction, including bone lesions and fractures, was evaluated by CT, MR, or PET-CT. In detail, imaging 1, 2, 3, and ≥4 osteolytic lesions were recorded as 1, 2, 3, and 4, respectively; and the presence or absence of fractures was recorded as 1 and 0, respectively.

The relationship between serum IL-17A levels and the BOE was determined. MM diagnosis was performed according to the new IMWG symptomatic MM criteria (1), and the efficacy evaluation was based on MM National Comprehensive Cancer Network (NCCN) clinical practice guidelines for 2020 (27).

Whether the serum IL-17A level was related to the patients’ clinical outcomes was further investigated by multivariate regression analysis. The ROC curve was used to assess the predictive value of serum IL-17A for the BOE. The PFS and OS were observed within 30 months.

IL-17A testing methods were as follows: serum concentrations of the above seven IL-17A were quantified by the Cytometry Bead Array (CBA) kit BD CBA Human TH17 Cytokine Kit (BD Biosciences, San Jose, CA, USA). The minimum and maximum detection limits for all seven cytokines were 0.01 and 5,000 pg/ml, respectively.

### Statistical analysis

The continuous data were expressed as mean ± standard deviation, and the categorical variables were expressed as numbers or percentages. Student’s t-test, chi-square test, or Mann–Whitney U test was used to assess the difference between the two groups.

A multivariate regression model adjusted by potential confounding factors was applied to estimate the serum IL-17A level and bone disease as well as fracture. Bone destruction in the skull, scapula, vertebrae, sternum/clavicle, ilium/pelvis/sacrum/pubis, and femur/humeral area detected by MR/CT/PET-CT was scored as 1 point. The total score of the bone-damage area was recorded as bone lesion level. Wherever detected, a fracture was recorded as 1. The violin plot shows the difference in bone disease and fractures with different serum IL-17A levels.

A multivariate regression model adjusted by confounding factors was used to estimate the independent relationship between serum IL-17A levels and the BOE (in statistical analysis, the best curative effect ≤complete response (≤CR) was labeled as 1, very good partial response (VGPR) as 2, partial response (PR) as 3, and stable disease (SD) as 4). A generalized additive model and smooth curve fitting were used to study the cross-sectional relationship between serum IL-17A levels and the BOE. ROC curve analysis was performed to evaluate the predictive value of serum IL-17A levels for the BOE. The area under the curve (AUC) of serum IL-17A was calculated and labeled as AUC. The Kaplan–Meier K-M curve further described the relationship between different levels of serum IL-17A and PFS or OS in different MM patient populations.

All probabilities were two-tailed. Empower Stats (www.empowerstats.com; X&Y Solution, Inc., Boston, MA, USA) and R software (http://www.r-project.org) were used to perform the statistics. A p-value <0.05 was considered significant.

## Results

**Figure 1:**
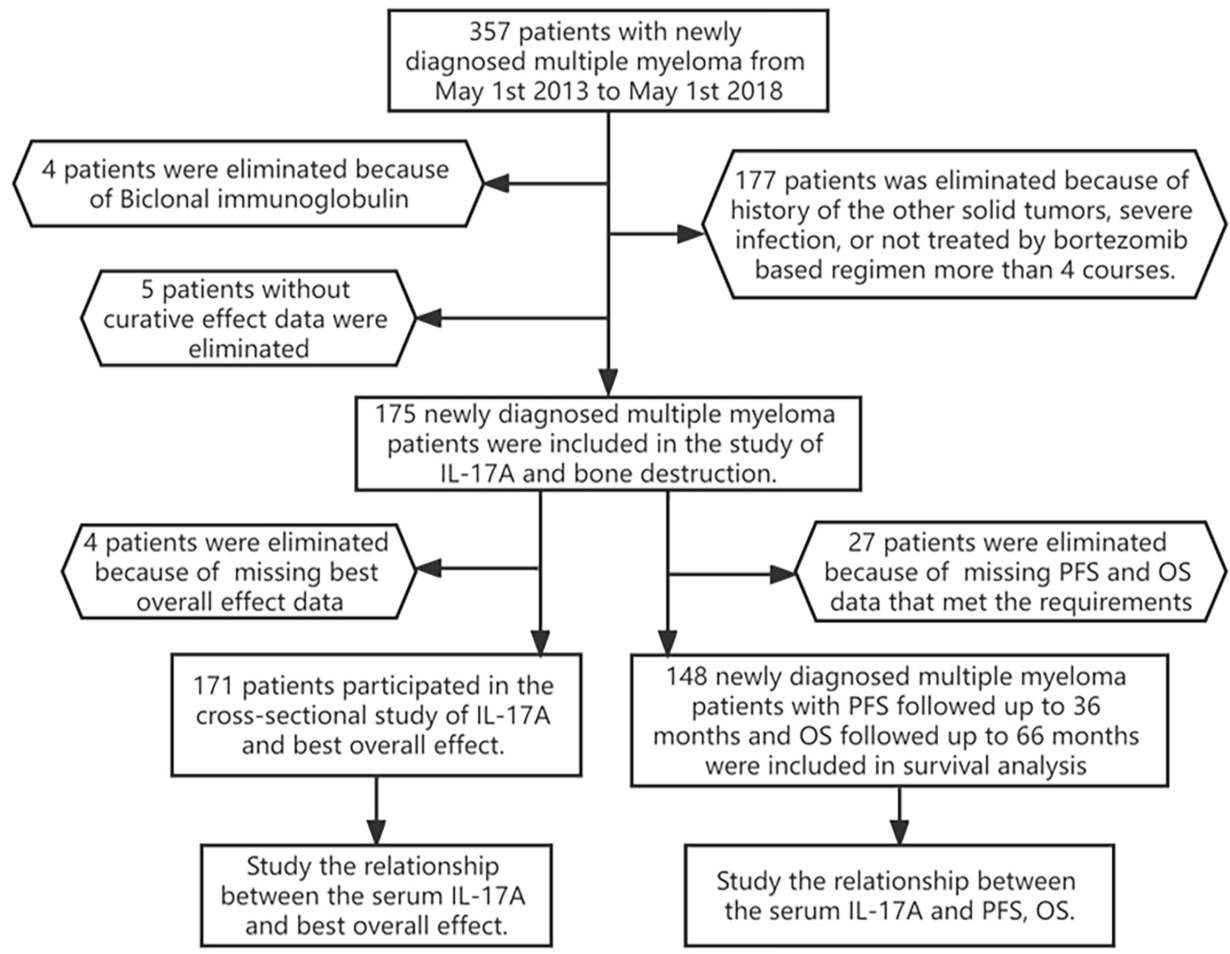
Flowchart of participants. A total of 175 patients participated in the cross-sectional study of IL-17A and bone destruction; 171 patients participated in the study of IL-17A and the best overall effect; 148 newly diagnosed multiple myeloma patients with PFS/OS were followed up for 30 months. PFS, progression-free survival; OS, overall survival.

From May 2013 to May 2018, among the 357 newly diagnosed MM patients treated in our department, 182 patients were excluded after the screening process (**Figure 1**), and 175 newly diagnosed MM patients were finally included in this study. A total of 171 MM patients were included in the cross-sectional study of the relationship between serum IL-17A and BOE, and 148 MM patients were included in the longitudinal study of the relationship between serum IL-17A and PFS/OS. See **Figure 1** for the specific inclusion and exclusion criteria.

### Serum IL-17A levels in multiple myeloma patients were independently associated with bone disease and fracture

The 175 MM patients included in this study had an average age of 62.36 ± 8.76 (76 of them, 43.43% were male) and a median of 0.10 pg/ml IL-17A, 104 (59.43%) exhibited baseline bone lesions at the time of initial onset, and 38 (21.84%) had fractures. The baseline characteristics of the study population are listed in **Table 1**.

**Table 1:** Demographics of patients with MM.

NDMM			
N	175	Age	62.36 ± 8.76(40.00–80.00)
Gender		Diabetes	14 (8.00%)
Female	99 (56.57%)	DS stage	
Male	76 (43.43%)	1	15 (8.57%)
Hypertension	61 (34.86%)	2	17 (9.71%)
Immunoglobulin type		3	143 (81.71%)
NA	36 (20.57%)	Creatinine	
IgG	79 (45.14%)	<177 μmol/L	173 (98.86%)
IgA	50 (28.57%)	≥177 μmol/L	2 (1.14%)
IgD	10 (5.71%)	IL-2 (pg/ml)	0.10 (0.01–11.74)
Light chain type		IL-4 (pg/ml)	0.10 (0.01-6.01)
NA	1 (0.57%)	IL-6 (pg/ml)	4.20 (0.10–1657.79)
κ	83 (47.43%)	IL-10 (pg/ml)	0.63 (0.01–49.26)
λ	91 (52.00%)	TNF-α (pg/ml)	0.10 (0.01–108.90)
ISS		IFN-γ (pg/ml)	0.10 (0.01–17.61)
1	57 (32.57%)	IL-17A (pg/ml)	0.10 (0.01–21.02)
2	51 (29.14%)	Bone lesion	
3	67 (38.29%)	0	71 (40.57%)
RISS		1	28 (16.00%)
NA	10 (5.85%)	2	24 (13.71%)
1	19 (11.11%)	3	30 (17.14%)
2	95 (55.56%)	4	22 (12.57%)
3	47 (27.49%)	Fracture	38 (21.84%)

Results in table: mean + SD/N(%)/median (min–max).

MM, multiple myeloma; NDMM, newly diagnosed multiple myeloma; ISS, International Staging System; RISS, revised ISS; DS, Durie–Salmon.

The results of single factor analysis showed that the serum IL-17A was related to the baseline bone lesion of new-onset MM patients and fracture. Since many factors may influence the BOE of MM patients, we conducted a multivariate regression analysis based on potentially related factors to further clarify the relationship between serum IL-17A and bone destruction in MM patients. The multivariate regression analysis indicated that serum IL-17A levels were independently correlated with baseline bone lesions in MM patients (β = 0.15; 95% CI (0.09, 0.21); p < 0.0001) and fractures (OR = 1.18; 95% CI (1.06, 1.31); p < 0.0001) (**Table 2**). When grouped according to the median value of serum IL-17A (0.1 pg/ml), the results showed that MM patients with IL-17A > 0.1 pg/ml had more severe bone lesions than MM patients with IL-17A ≤ 0.1 pg/ml (β = 1.60; 95% CI (1.06, 2.13); p < 0.0001) and a higher incidence of fracture (OR = 6.77; 95% CI (2.61, 17.55); p < 0.0001) among the participants (**Table 2**).

**Table 2:** Multiple regressions analyses between serum IL-17A and bone lesion/fracture in different models.

Models		Fully adjusted model (β, 95% CI, p)
Bone lesion	IL-17A	0.15 (0.09, 0.21) <0.0001
	≤0.1 pg/ml	0
	>0.1 pg/ml	1.60 (1.06, 2.13) <0.0001
**Multifactor adjusted linear regression**		
Models		**Fully adjusted model (OR, 95% CI, p)**
Fracture	IL-17A	1.18 (1.06, 1.31) 0.0029
	≤0.1 pg/ml	1
	>0.1 pg/ml	6.77 (2.61, 17.55) <0.0001

Multifactor adjusted logistic regression.

Fully adjusted model adjusted for gender, age, hypertension, diabetes, light chain type, creatinine, and RISS stage.

RISS, revised International Staging System.

**Figure 2:**
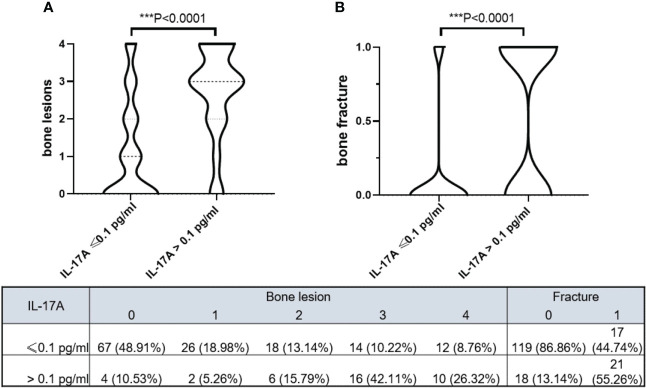
Violin plots show bone lesion levels and bone fracture in different serum IL-17A groups. **(A)** MM patients with serum IL-17A > 0.1 pg/ml had higher bone lesion levels (p < 0.0001). **(B)** MM patients with serum IL-17A > 0.1 pg/ml had more bone fracture (p < 0.0001). The model was adjusted for age, sex, hypertension history, diabetes history, immunoglobulin type, light chain type, RISS, and serum creatinine. Bone destruction in the skull, scapula, vertebrae, sternum/clavicle, ilium/pelvis/sacrum/pubis, and femur/humeral area detected by MR/CT/PET-CT scored 1 point, and the total score of the bone-damage area was recorded as bone lesion level. Wherever detected, a fracture was recorded as 1. MM, multiple myeloma; RISS, revised International Staging System. *** means P<0.001.

The violin plot chart intuitively showed that MM patients with IL-17A > 0.1 pg/ml had more severe bone lesions (**Figure 2A**) and fractures (**Figure 2B**). MM patients with IL-17A ≤ 0.1 pg/ml mainly had 0–2 level of bone lesions and no baseline fracture. However, patients with IL-17A > 0.1 pg/ml mainly suffered 2–4 bone lesion level and a higher baseline fracture rate.

### The serum IL-17A level of multiple myeloma patients was independently correlated with the best overall effect, especially obvious in the light chain and IgA subgroups

Among the 171 newly diagnosed MM patients, 83 (48.54%), 34 (19.88%), 49 (28.65%), and 5 (2.9%) had the BOE with CR, VGPR, PR, and SD, respectively. In terms of factor analysis, the older the age, the worse the BOE (average age CR vs. VGPR vs. PR vs. SD: 61.14 ± 8.87 vs. 61.76 ± 9.09 vs. 64.45 ± 7.94 vs. 70.40 ± 7.50, p = 0.032); female patients were better than male patients (female vs. male CR, VGPR, PR, SD: 39 (46.99%) vs. 44 (53.01%), 19 (55.88%) vs. 15 (44.12%), 37 (75.51%) vs. 12 (24.49%), and 3 (60.00%) vs. 2 (40.00%), respectively; p = 0.016) (**Table 3**).

**Table 3:** Demographics of patients with MM in different efficacy.

	Best overall effect (BOE)				
	CR	VGPR	PR	SD	p-Value
N	83 (48.54%)	34 (19.88%)	49 (28.65%)	5 (2.9%)	
Age	61.14 ± 8.87	61.76 ± 9.09	64.45 ± 7.94	70.40 ± 7.50	0.032
Gender					0.016
Female	39 (46.99%)	19 (55.88%)	37 (75.51%)	3 (60.00%)	
Male	44 (53.01%)	15 (44.12%)	12 (24.49%)	2 (40.00%)	
Hypertension	29 (34.94%)	12 (35.29%)	17 (34.69%)	1 (20.00%)	0.923
Diabetes	5 (6.02%)	1 (2.94%)	7 (14.29%)	0 (0.00%)	0.181
Immunoglobulin type					0.002
NA	26 (31.33%)	4 (11.76%)	4 (8.16%)	1 (20.00%)	
IgG	23 (27.71%)	17 (50.00%)	33 (67.35%)	4 (80.00%)	
IgA	28 (33.73%)	11 (32.35%)	10 (20.41%)	0 (0.00%)	
IgD	6 (7.23%)	2 (5.88%)	2 (4.08%)	0 (0.00%)	
Light chain type					0.072
κ	32 (38.55%)	22 (64.71%)	23 (46.94%)	3 (60.00%)	
λ	51 (61.45%)	12 (35.29%)	26 (53.06%)	2 (40.00%)	
DS stage					0.586
1	7 (8.43%)	3 (8.82%)	5 (10.20%)	0 (0.00%)	
2	7 (8.43%)	6 (17.65%)	3 (6.12%)	0 (0.00%)	
3	69 (83.13%)	25 (73.53%)	41 (83.67%)	5 (100.00%)	
Creatinine					0.543
<177 μmol/L	81 (97.59%)	34 (100.00%)	49 (100.00%)	5 (100.00%)	
≥177 μmol/L	2 (2.41%)	0 (0.00%)	0 (0.00%)	0 (0.00%)	
ISS					0.275
1	32 (38.55%)	10 (29.41%)	14 (28.57%)	0 (0.00%)	
2	23 (27.71%)	13 (38.24%)	12 (24.49%)	3 (60.00%)	
3	28 (33.73%)	11 (32.35%)	23 (46.94%)	2 (40.00%)	
RISS					0.083
1	12 (14.63%)	1 (2.94%)	5 (10.64%)	0 (0.00%)	
2	46 (56.10%)	26 (76.47%)	19 (40.43%)	3 (75.00%)	
3	19 (23.17%)	7 (20.59%)	19 (40.43%)	1 (25.00%)	
IL-2 (pg/ml)	0.10 (0.01–2.55)	0.10 (0.10–11.74)	0.10 (0.01–4.89)	0.10 (0.10–1.70)	0.438
IL-4 (pg/ml)	0.10 (0.01–2.69)	0.10 (0.01–2.65)	0.10 (0.01–6.01)	0.10 (0.10–2.77)	0.266
IL-6 (pg/ml)	5.77 (0.10–1657.79)	3.61 (0.10–400.02)	3.09 (0.10–1049.52)	5.65 (4.11–26.50)	0.68
IL-10 (pg/ml)	0.60 (0.01–13.50)	0.46 (0.10–7.24)	0.76 (0.01–49.26)	2.94 (0.10–8.58)	0.384
TNF-α (pg/ml)	0.10 (0.01–87.15)	0.10 (0.10–108.90)	0.10 (0.01–12.29)	0.57 (0.10–2.10)	0.228
IFN-γ (pg/ml)	0.10 (0.01–17.61)	0.10 (0.10–9.17)	0.10 (0.01–11.30)	0.10 (0.10–4.76)	0.762
IL-17A (pg/ml)	0.10 (0.01–20.49)	0.10 (0.01–16.12)	0.10 (0.01–17.80)	1.67 (0.10–21.02)	0.052
Therapeutic regimen					0.348
PD	14 (17.07%)	3 (8.82%)	12 (24.49%)	3 (60.00%)	
PCD	47 (57.32%)	24 (70.59%)	26 (53.06%)	1 (20.00%)	
PAD	12 (14.63%)	5 (14.71%)	5 (10.20%)	1 (20.00%)	
PTD	2 (2.44%)	1 (2.94%)	3 (6.12%)	0 (0.00%)	
PRD	7 (8.54%)	1 (2.94%)	3 (6.12%)	0 (0.00%)	
ASCT					0.019
NO	65 (78.31%)	31 (91.18%)	47 (95.92%)	5 (100.00%)	
Yes	18 (21.69%)	3 (8.82%)	2 (4.08%)	0 (0.00%)	

Results in table: mean + SD/N(%)/median (min–max).

ISS, International Staging System; RISS, revised ISS; PD, bortezomib and dexamethasone; PCD, bortezomib, cyclophosphamide and dexamethasone; PAD, bortezomib, adriamycin and dexamethasone; PTD, bortezomib, thalidomide and dexamethasone; PRD, bortezomib, lenalidomide and dexamethasone; ASCT, autologous stem cell transplantation; CR, complete remission; PR, partial remission; VGPR, very good PR; SD, stable disease; BOE, best overall efficacy.

The correlation analysis showed that old age (β = 0.21, p = 0.0007) and non-light chain type (β_IgG_ = 0.81, p < 0.0001; β_IgA_ = 0.2, p = 0.294; β_IgD_ = 0.17, p = 0.587) were associated with a poorer BOE, while male gender (β = −0.23, p = 0.0031) and autologous stem cell transplantation (ASCT) (β = −0.64, p = 0.0018) were associated with a better BOE. A multivariate regression analysis adjusted by the above potential related factors showed that the IL-17A serum level was independently correlated with the BOE (the p-values were 0.043, 0.028, and 0.037, **Table 4A**). In particular, the stratified multivariate regression analysis based on immunoglobulin classification showed that in the light chain type (in the three progressive models, the p-values were 0.004, 0.002, and 0.009) and IgA patients (in the three progressive models, the p-values were 0.156, 0.130, and 0.0477), serum IL-17A was independently correlated with the BOE, and the serum IL-17A did not show an independent correlation with the BOE in the IgG and IgD types (**Table 4B**).

**Table 4A:** Multiple regressions analyses between serum IL-17A and best overall effect (BOE) in different models.

Variable	Fully adjusted model (β, 95% CI, p)
IL-17A	0.04 (0.00, 0.08) 0.0371
≤0.1 pg/ml	0
>0.1 pg/ml	0.37 (0.01, 0.74) 0.0456

Multifactor adjusted linear regression.

Fully adjusted model adjusted for gender, age, hypertension, diabetes, immunoglobulin type, light chain type, creatinine, and RISS stage, therapeutic regimen, and autologous stem cell transplantation.

RISS, revised International Staging System.

**Table 4B:** Multiple regressions analyses between serum IL-17A and best overall effect (BOE) in different immunoglobulin types.

Variable	Fully adjusted model (β, 95% CI, p)
Light chain	0.12 (0.04, 0.20) 0.0086
IgG	NA
IgA	0.09 (0.00, 0.17) 0.0477
IgD	NA

Multifactor adjusted linear regression.

Fully adjusted model adjusted for gender, age, hypertension, diabetes, light chain type, creatinine, RISS stage, therapeutic regimen, and autologous stem cell transplantation.

RISS, revised International Staging System. NA, not applicable.

**Figure 3:**
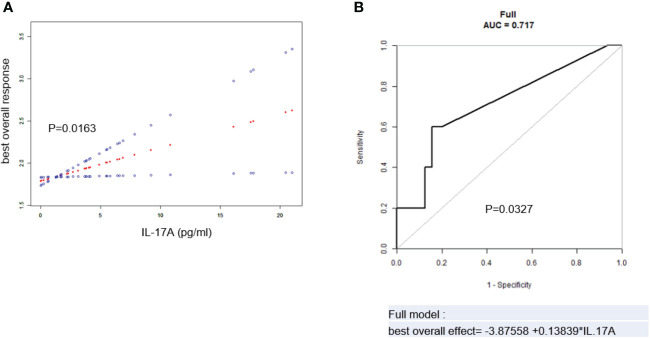
**(A)** Spline smoothing curve for the cross-sectional correlation between serum IL-17A and best overall effect (BOE) (p = 0.0163). **(B)** Predictive model and ROC analysis for IL-17A and BOE (p = 0.0327). The model was adjusted for age, sex, hypertension history, diabetes history, immunoglobulin type, light chain type, RISS, serum creatinine, therapeutic regimen, and autologous stem cell transplantation. BOE ≤ CR was labeled as 1, VGPR as 2, PR as 3, and SD as 4. ROC, receiver operating characteristic; RISS, revised International Staging System; CR, complete remission; VGPR, very good partial remission; PR, partial remission; SD, stable disease.

After adjustment of the above potential related factors, it could be seen from the smooth curve analysis that the high serum IL-17A level was positively correlated with poorer BOE (p = 0.0163, **Figure 3A**).

The ROC analysis of IL-17A and the BOE was performed to evaluate the predictive value of serum IL-17A level in MM patients for the BOE. After adjustment of potentially related factors, the model showed that the AUC was 0.717 (p = 0.0327, **Figure 3B**), suggesting that the serum IL-17A level had a certain predictive value for the BOE in MM patients.

### In light chain multiple myeloma patients, high serum IL-17A had poorer progression-free survival and overall survival

In order to evaluate the predictive value of serum IL-17A level in the prognosis of MM patients, the baseline serum IL-17A level was divided into two groups by a median of 0.1 pg/ml, and the clinical results were compared (**Table S1**). A total of 148 newly diagnosed MM patients were included in the observational cohort. In the IL-17A ≤ 0.1 and IL-17A > 0.1 groups, the average age was 62.33 ± 8.81 and 64.96 ± 8.33, there were 49 (41.53%) and 13 (43.33%) male patients, and the median PFS was 19.50 and 17.50 months, and the median OS was 26.03 and 24.10 months, respectively.

**Figure 4:**
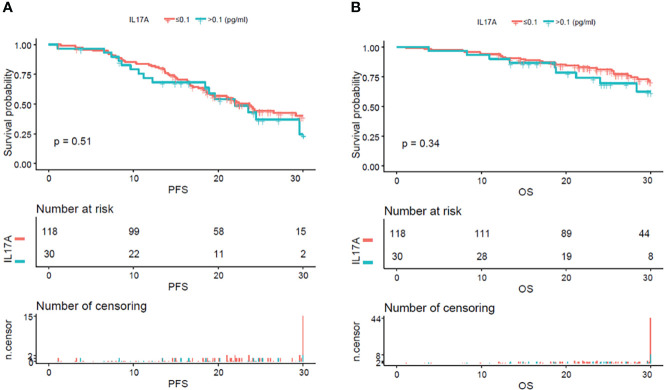
Kaplan–Meier curves of progression-free survival (PFS) and overall survival (OS) according to mean serum IL-17A levels. There were no obvious difference in PFS **(A)** (p = 0.51) and OS **(B)** (p = 0.34) between the patients with serum IL-17A ≥ 0.1 pg/ml and IL-17A <0.1 pg/ml.

**Figure 5:**
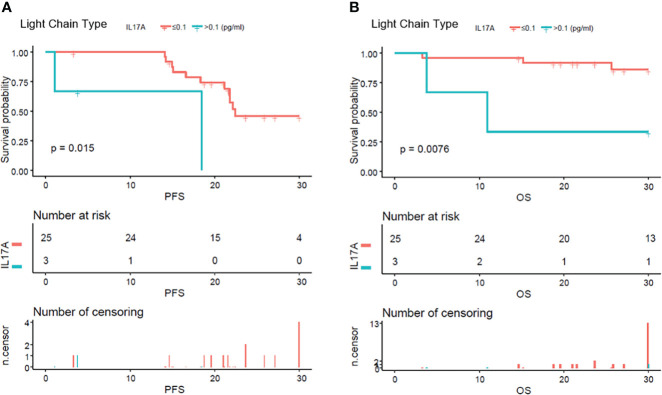
Kaplan–Meier curves of progression-free survival (PFS) and overall survival (OS) according to mean serum IL-17A levels in pure light chain type multiple myeloma patients. Compared with patients with serum IL-17A > 0.1 pg/ml, PFS **(A)** (p = 0.015) and OS **(B)** (p = 0.0076) were significantly lower in patients with serum IL-17A ≤ 0.1 pg/ml.

**Figure 6:**
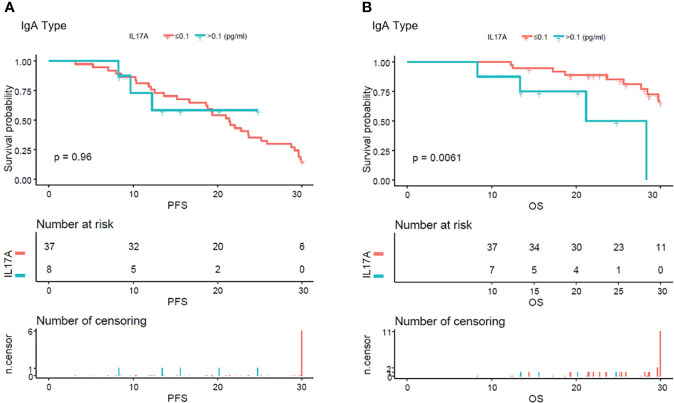
Kaplan–Meier curves of progression-free survival (PFS) and overall survival (OS) according to mean serum IL-17A levels in IgA type multiple myeloma patients. **(A)** There were no obvious difference in between the patients with serum IL-17A ≤ 0.1 pg/ml and IL-17A > 0.1 pg/ml (p = 0.96). **(B)** Compared with patients with serum IL-17A > 0.1 pg/ml, OS was significantly lower in patients with serum IL-17A ≤ 0.1 pg/ml (p = 0.0061).

From the analysis of the K-M curve, it could also be seen that there was no significant difference in PFS and OS between MM patients with serum IL-17A > 0.1 and MM patients with IL-17A ≤ 0.1 (**Figures 4A, B**). The K-M curve analysis based on immunoglobulin stratification suggested that, in the light chain subgroup, compared with patients with serum IL-17A ≤ 0.1, patients with IL-17A > 0.1 had poorer PFS (p = 0.015; **Figure 5A**) and OS (p = 0.0076; **Figure 5B**). In the IgA type subgroup, patients with IL-17A > 0.1 had poorer OS (p = 0.0061; **Figure 6B**), but there was no significant difference in PFS (p = 0.96; **Figure 6A**). IL-17A level also had no significant effect on PFS/OS in IgG and IgD type subgroups based on our data (**Supplementary Figures 1**, **2**).

## Discussion

Myeloma-mediated osteolytic lesions are the hallmark of MM. About 80% of newly diagnosed patients with MM present bone destruction (28), which seriously affects the morbidity and mortality and in turn the quality of life of patients (29). In this study, we found that the MM patients’ serum IL-17A level was independently associated with bone lesions and fractures, and the high level of serum IL-17A (>0.1 pg/ml) was related to severe baseline bone disease, which was consistent with the results of previous clinical and experimental research performed by Noonan et al. (20). Their results provide a possible mechanism for our result of the association between IL-17A level and fracture/bone lesion. In addition, IL-17A can stimulate immature dendritic cells (DCs) in MM to elicit osteoclast-like differentiation and enhance lytic bone lesions (30). We revealed the correlation between the serum IL-17A and the bone lesions of MM patients at the first diagnosis. The follow-up of serum IL-17A level, remission, or aggravation of bone lesions can be further tracked in the future to evaluate the predictive value of IL-17A in the occurrence or reduction of bone lesions.

In MM, many prognostic and predictive biomarkers are of high value in predicting therapeutic efficacy, as well as indicating PFS and OS. Translocations, copy number abnormalities and mutations at the gene level, minimal residual disease (MRD) measured by flow cytometry and next-generation sequencing (NGS) detection, blood biopsy detection of circulating tumor cells (CTCs), circulating tumor DNA (ctDNA), and baseline clinical characters have gradually become advanced biomarkers (31–33). However, these indicators are still not perfect, and certain examinations are invasive or costly. Therefore, more biomarkers are needed to better predict the therapeutic efficacy and prognosis and help to provide stratified treatment for MM patients. Cytokines play a vital role in the tumor microenvironment and affect the occurrence and development of MM. Previous studies have shown that a variety of cytokines are closely related to prognosis. For example, high levels of IL-6 in the serum of MM patients are associated with poor prognosis (34), and high levels of IL-10 indicate shorter PFS and OS in MM patients (35). Previous studies have demonstrated that, among newly diagnosed MM patients, those with normal Th17 cell proportion can attain CR the fastest and have the longest PFS (36); a higher ratio of IL-27:IL-17 in the bone marrow is related to longer PFS (24). Compared with patients in refractory relapse and partial remission, MM patients in complete remission have lower IL-17 levels at the time of initial treatment (37). Gu also evaluated the relationship between IL-17A level and the curative effect and prognosis of newly diagnosed MM patients in China from previous reports. They found that patients with lower IL-17A levels had a higher OR rate, and those with serum IL-17A ≥ 4 pg/ml had shorter OR rates, PFS, and OS as compared to those with serum IL-17A < 4 pg/ml (38). The above results of these published articles are consistent with our findings.

Concerning the relationship between IL-17 serum levels and the MM patient’s survival and therapeutic response, the possible mechanisms are that elevated IL-17 produced by Th17 cells promotes myeloma cell growth and inhibits immune function by IL-17A receptor (IL-17RA) expressed on tumor cells (21, 38). Wang et al. found that IL-17A increased multiple myeloma cell viability by positively regulating Syk expression (22). Many factors regulate the level of IL-17A in MM patients during the development of MM disease. For example, IL-6, TGF-β, and IL-23 can promote the differentiation of Th17 lymphocytes, and some microRNAs like miR-21, as well as intestinal microecology, can also drive the differentiation of Th17 and induce the production of IL-17A (39, 40). It is clear that the production of IL-17A in MM is affected by complex factors. For example, mature DCs can induce the proliferation of autologous Th17 cells and increase the secretion of IL-17A (41). During the treatment of MM patients, changes in certain factors are likely to change the level of IL-17A, thereby affecting the prognosis of MM. More in-depth mechanisms and clinical translation applications remain to be explored in further scientific research.

IL-17A is a founding member of the IL-17 cytokine family, and other members include IL-17B, IL-17C, IL-17D, and IL-17E (5). Their functions partially overlap with those of IL-17A, although they have not been thoroughly investigated. A variety of immune and inflammatory cells produce IL-17A, especially Th17 cells, a subset of CD4+ T cells (42). TH17, as IL-17A secreting cells, plays an important role in regulating the level of IL-17A. The change in TH17 content is also related to the level of IL-17A. Th17 acts as a subset of osteocyte-destroying helper T cells, linking T-cell activation and bone destruction (43). Recent studies have also shown that Th17 secretes IL-17A, which induces pyroptosis in osteoblasts *via* the NLRP3 inflammasome pathway *in vitro* (44). Th17 cells play a key role in the pathogenesis of bone destruction, inflammation, and autoimmune diseases, but their role in infiltrating and exerting in tumor cells is also attracting more attention (45). Su et al. found that tumor cells and tumor-derived fibroblasts produce a pro-inflammatory cytokine milieu that promotes Th17 cell recruitment, production, and expansion (46). Recently, Mucciolo et al. found that IL-17A shapes the transcriptional program of fibroblasts in pancreatic cancer and switches on their protumorigenic functions, with the frequency of Th17 higher in the first weeks of age in mice (47). In multiple myeloma, studies have suggested that Th17 cells are involved in the pathophysiological process of MM, and RORC overexpression is a sign of the accumulation of Th17 lymphocytes in the bone marrow of multiple myeloma (48). It was reported that overexpression of CCl20 and CCR6 was also involved in the recruitment of Th17 in the bone microenvironment of myeloma patients (49). Rossi et al. found that TH17 promotes the progression of multiple myeloma, and miR-21 antagonism abrogates its promoting functions (39). It was also reported that MM cells express IL-17 and found that IL-17/miR-192/IL-17R regulatory feedback loop was important in the progression of MM (50). In addition, microbiota-propelled Th17 promotes cancer progression (51), and there is evidence showing that microbiota-driven interleukin-17-producing cells synergize to accelerate multiple myeloma progression (40). These are also possible mechanisms to explain why the IL-17A level is related to the bone destruction, prognosis, and treatment response of MM patients.

This article leaves much to be desired. In the present work, we retrospectively analyzed the relationship between serum IL-17A and bone destruction, BOE, and prognosis. We found that high levels of IL-17A were related to severe bone lesions and fractures. However, the 95% CI range was large in the fracture section, which should be related to our small sample size. It is worth looking forward to further expanding the sample size to obtain better results. Meanwhile, it was shown that the lower the IL-17A level, the better the BOE after treatment. Especially in the light chain and IgA patients, IL-17A was independently related to the BOE. The ROC analysis also revealed that the IL-17A level could partially predict MM patients’ BOE. In terms of assessing the predictive value of serum IL-17A levels in the prognosis of MM patients, we could not establish that IL-17A was correlated with PFS and OS in the samples of all 148 participants. This may be related to our limited sample size and insufficient follow-up time. In particular, in the stratified analysis by immunoglobulin type, the amount of sample was smaller due to subgrouping. Clinical studies with more adequate sample sizes need to be further performed. Therefore, the conclusion has certain limitations. In addition, as a retrospective clinical study, our conclusions have limited application for direct clinical translation. Whether the monoclonal antibody of IL-17A is effective in the clinical treatment of MM patients, or whether it can synergize with other therapeutic drugs, remains to be proved by further clinical trials. We are also planning for relevant clinical trials. We looking forward to more definitive results that are helpful for clinical application.

## Conclusions

Our research demonstrated that the baseline serum IL-17A level of newly diagnosed MM patients in China was independently related to the degree of bone lesions and fractures. There was a linear relationship between high serum IL-17A levels and poor BOE. The baseline serum IL-17A level can predict the BOE in MM patients to a certain extent. We also found that MM patients with high serum IL-17A levels had poorer PFS and OS in the light chain subgroup and poorer OS in the IgA subgroup. These findings indicated that high serum IL-17A levels could be used as a clinical biomarker for bone destruction and poor prognosis in MM patients. Our findings also provided clinical data support for the use of monoclonal antibodies targeting IL-17A in the future.

## Data availability statement

The original contributions presented in the study are included in the article/**Supplementary Material**. Further inquiries can be directed to the corresponding authors.

## Ethics statement

This study was reviewed and approved by Ethical Inspection of the First Affiliated Hospital, College of Medicine, Zhejiang University. Written informed consent for participation was not required for this study in accordance with the national legislation and the institutional requirements.

## Author contributions

ZC and JH designed research and guided the project processing and the paper writing. MD performed the clinical data collection, analyzed the data, and wrote the paper. JZ collected the clinical data and wrote mainly the background and discussion parts of the article. QC, DH, HY, GZ, and XH provided the patient cases. All authors contributed to the article and approved the submitted version.

## Funding

This work was supported by the National Natural Science Foundation of China (82100212) and Zhejiang Provincial Natural Science Foundation Key Project (LZ22H160009).

## Acknowledgments

The authors are grateful to the other members of the Bone Marrow Transplantation Center, The First Affiliated Hospital, School of Medicine, Zhejiang University, for helpful discussions.

## Conflict of interest

The authors declare that the research was conducted in the absence of any commercial or financial relationships that could be construed as a potential conflict of interest.

## Publisher’s note

All claims expressed in this article are solely those of the authors and do not necessarily represent those of their affiliated organizations, or those of the publisher, the editors and the reviewers. Any product that may be evaluated in this article, or claim that may be made by its manufacturer, is not guaranteed or endorsed by the publisher.
