# Supplementary figures and images for: High serum IL-17A is associated with bone destruction in newly diagnosed multiple myeloma patients
Source: Front Oncol. 2022 Aug 31;12:936670. doi: 10.3389/fonc.2022.936670 (PMC9471080; doi:10.3389/fonc.2022.936670)

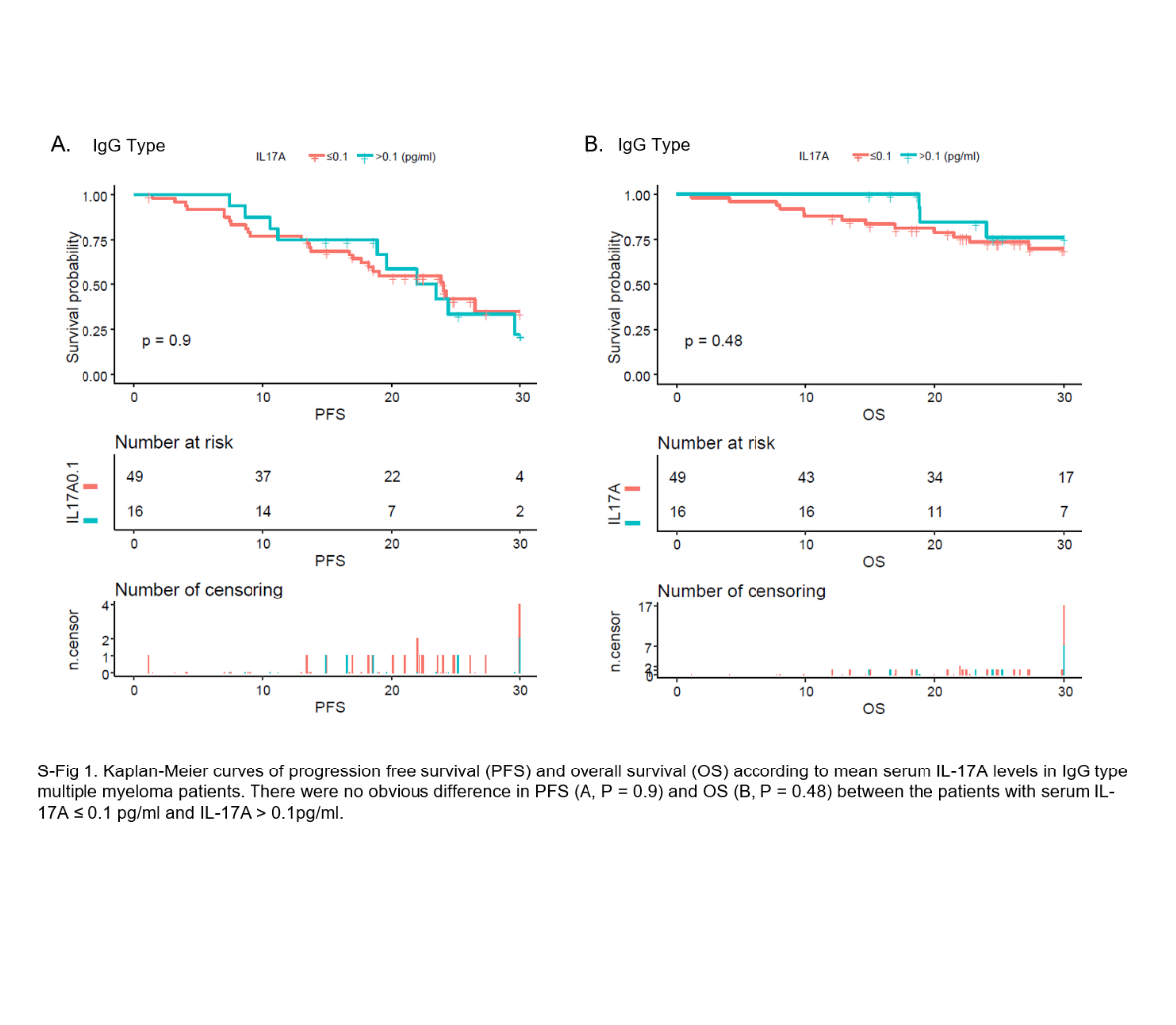

Supplement: Supplementary file 1 [file Image_1.jpeg]

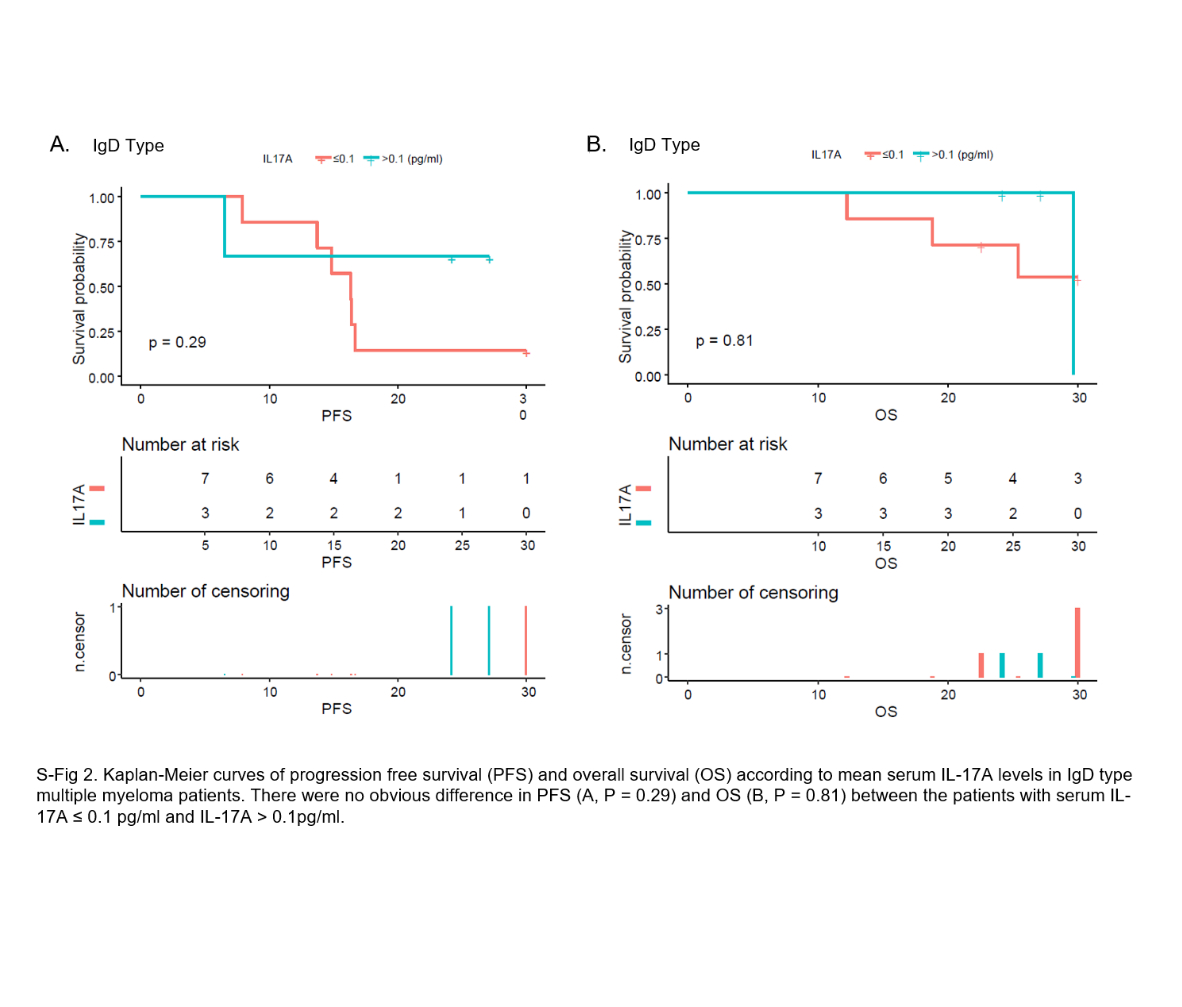

Supplement: Supplementary file 2 [file Image_2.jpeg]
